# Progress toward the eradication of dracunculiasis (Guinea worm disease).

**DOI:** 10.3201/eid0102.950205

**Published:** 1995

**Authors:** E Ruiz-Tiben, D R Hopkins, T K Ruebush, R L Kaiser

**Affiliations:** Global 2000 Project, The Carter Center of Emory University, Atlanta, Georgia, USA.


### Progress Toward the Eradication of Dracunculiasis (Guinea Worm Disease): 1994

Dracunculiasis, or guinea worm disease (GWD), is a disabling infection caused by the nematode parasite *Dracunculus medinensis*. The disease is endemic in India, Africa, and the Middle East. People become infected when they drink water containing tiny crustaceans, called copepods or "water fleas," that act as intermediate hosts of the organism and harbor infective larvae. When the ingested copepods are killed by the digestive juices in the stomach, the larvae are released and move to the small intestine. They penetrate the intestinal wall and migrate to the connective tissues of the thorax, where male and female larvae mature and mate 60 to 90 days after infection. Over the next year, female worms grow to maturity, reach a length of 70 cm or more (2–3 feet), and slowly migrate to the surface of the body. Worms emerge from the lower extremities in about 90% of cases, but they can also appear in the upper extremities, the trunk, buttocks, genitalia, or other parts of the body.

Infected persons remain asymptomatic for approximately a year after infection when the mature female worm approaches the skin and forms a painful papule in the dermis. This papule can become a blister within 24 hours or may enlarge for several days before becoming a blister. Eventually it ruptures, exposing the worm. Shortly before the skin lesion forms, pronounced systemic symptoms may occur, including erythema and urticarial rash with intense pruritus, nausea, vomiting, diarrhea, and dizziness. On contact with fresh water, a loop of the worm's uterus opens and discharges a swarm of motile larvae. This process may be repeated, if the lesion is resubmerged in water, until the entire brood of larvae is discharged. Larvae ingested by copepods mature in the body cavity of the intermediate host in about 2 weeks. Stagnant sources of drinking water such as ponds, cisterns, pools in dried-up river beds, temporary hand-dug wells, and step-wells commonly harbor populations of freshwater copepods and are the usual sites where the infection is transmitted.

As the worm emerges through the skin lesion, the affected person pulls it out slowly and carefully, usually by winding a few centimeters each day on a stick. This very painful process may last many weeks. Pain and other symptoms may lessen with the rupture of the blister, but at this time pyogenic organisms often invade the superficial lesion and worm tract and aggravate the condition. If the worm breaks during traction, an intense inflammatory reaction occurs, with pain, swelling, and cellulitis along the worm track. Infected persons are often incapacitated for several weeks, or for months, if complications caused by secondary bacterial infections ensue.

Infected persons do not develop immunity, and there is no cure for GWD. Pain from emerging worms may be relieved by applying wet compresses to the lesion or by immersing the lesion in a container of water, and then safely disposing of the released larvae. Placing an occlusive bandage on the wound to keep it clean prevents the patient from contaminating sources of drinking water. Once the worm appears, oral medications to relieve the associated pain and topical antiseptics or antibiotic ointment to minimize the risk for secondary infections allows the worm to be removed by gentle traction over a number of days.

The World Health Organization (WHO) has targeted GWD for eradication by the end of 1995. A coalition of agencies, institutions, and organizations are providing technical and financial assistance to national eradication programs.

An understanding of the factors that contribute to the emergence of dracunculiasis provides the basis for the current elimination program. GWD can be eradicated for several reasons: 1) there is no human carrier beyond the 1-year incubation period; 2) there is no known animal reservoir; 3) detection of patent infections (i.e., worms protruding from skin lesions) is an easy way to assess the presence of the disease in communities, and protrusion of the worm is required for transmission; 4) transmission of the disease is markedly seasonal, facilitating the timing and effectiveness of surveillance and control interventions, including containment of cases; 5) the methods for controlling transmission are simple, and 6) the disease is well recognized by the local population in areas where it is endemic.

The overall strategy of national GWD eradication programs includes three operational phases: l) conducting baseline surveys to identify villages where the disease is endemic; 2) training village-based health workers to use case registries for monthly surveillance and to implement control interventions in affected communities; and 3) using case-containment, i.e., prompt detection of all remaining cases (before or within 24 hours of worm emergence), treatment of the lesions to prevent transmission, and close supervision of village-based health workers to ensure that each case has been properly contained (1).

The thrust of control interventions is to educate affected persons about the origin of the disease and about the measures they and their communities can take to prevent it. Affected households are provided with cloth filters and taught how to safely use them to remove the copepods from water. Household members are also taught that those with emerging worms should be kept from entering sources of drinking water. Other control interventions include providing safe drinking water to affected villages and selectively using the insecticide temefos to reduce copepod populations.

In 1986, an estimated 3.32 million cases of GWD occurred in Africa (2). That same year, only 22,610 cases of GWD were reported from India, the only affected country with a national eradication campaign then underway. Worldwide cases reported to WHO have declined from 781,219 in 1988 to 221,055 in 1993 (3). By 1990, 9 of the 19 affected countries had initiated eradication programs and had conducted baseline surveys to assess the extent of GWD (Figure 1, Table 1). At the end of 1994, all 19 countries were actively combating the diseases, and the provisional number of cases reported to WHO was 164,750 (Figure 2, Table 2). The number of affected villages was reduced from more than 23,000 in 1993 to fewer than 10,000; 52% of the affected villages were in the case-containment phase.

In 1994, Pakistan had no cases (4). (Pakistan is the first country where GWD was endemic during the 1980s to have eliminated indigenous transmission of the disease for 1 year.) Yemen reported small foci of disease transmission for the first time in several years, and Sudan reported 28,899 GWD cases during active village-based searches, and through its passive surveillance system (5). In early March 1995, Sudan revised the number of cases reported to WHO for 1994 to 53,092 cases. Although

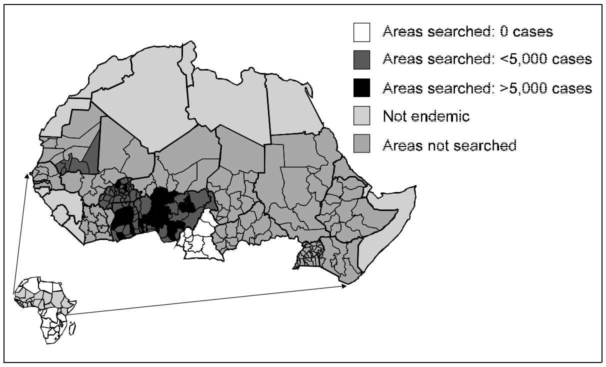

Figure 1. Status of dracunculiasis eradication in Africa: 1990.

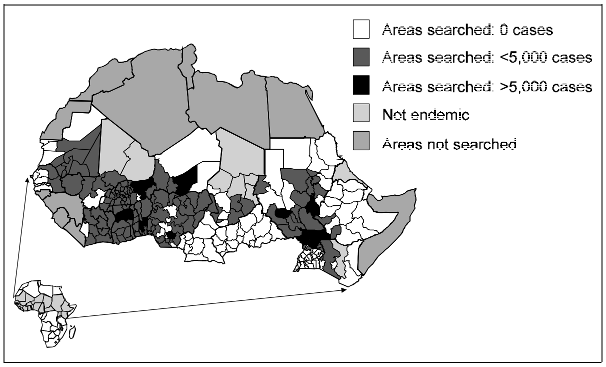

Figure 2. Status of dracunculiasis eradication in Africa: 1994.

**Table 1. Dracunculiasis cases reported, 1990***

| Country | Number of cases |
|---|---|
| Nigeria | 394,082 |
| Ghana | 117,034 |
| Burkina Faso | 42,187 |
| Benin | 37,414 |
| Mauritania | 8,036 |
| India | 4,798 |
| Togo | 3,042 |
| Cameroon | 742 |
| Pakistan | 160 |
| Chad† | – |
| Côte d'Ivoire† | – |
| Ethiopia† | – |
| Kenya† | – |
| Mali† | – |
| Niger† | – |
| Senegal† | – |
| Sudan† | – |
| Uganda† | – |
| Yemen† | – |

*Cases reported to the World Health organization from countries completing national case searches or from active village-based surveillance.
† No information available at this time.

**Table 2. Dracunculiasis cases reported, 1994***

| Country | Number of cases |
|---|---|
| Sudan | 53,092 |
| Nigeria | 39,774 |
| Niger | 23,568 |
| Uganda | 10,409 |
| Ghana | 8,432 |
| Burkina Faso | 6,859 |
| Mali | 5,396 |
| Togo | 5,045 |
| Mauritania | 5,029 |
| Côte d'Ivoire | 4,700 |
| Benin | 3,440 |
| Ethiopia | 1,252 |
| Chad | 640 |
| India | 371 |
| Senegal | 186 |
| Yemen | 74 |
| Kenya | 37 |
| Cameroon | 30 |
| Pakistan | 0 |

*Provisional data reported to the World Health Organization from countries completing national case searches or from active village-based surveillance. Sudan's report to WHO includes cases reported by the Passive Surveillance System.

the number of cases reported by Sudan may not be precise, their relative magnitude likely reflects that the incidence of disease there exceeds that reported by any of the other affected countries.

The global GWD eradication campaign faces two major challenges in 1995: to complete implementation of the case-containment strategy and other control interventions, such as insecticide use, in villages of all affected countries and to mobilize greater public support in these countries. The countries that pose the greatest eradication challenge are Sudan, Niger, and Nigeria. However, even in the countries with the fewest cases, markedly tighter control measures will be required to completely interrupt transmission of GWD by the end of 1995. Success of the eradication campaign depends on continued funding and on the ability of national programs and collaborating agencies and organizations to complete the needed surveillance and control interventions.

**Ernesto Ruiz-Tiben, Donald R. Hopkins, Trenton K. Ruebush,* and Robert L. Kaiser**
Global 2000 Project, The Carter Center of Emory University, Atlanta, Georgia, USA
*National Center for Infectious Diseases, Centers for Disease Control and Prevention, Atlanta, Georgia, USA

### References

1. Hopkins DR, Ruiz-Tiben E. Strategies for dracunculiasis eradication. Bull WHO 1991;69:533-40.
2. Watts S. Dracunculiasis in Africa in 1986: its geographic extent, incidence, and at-risk population. Am J Trop Med Hyg 37:119-25.
3. WHO. Dracunculiasis—global surveillance summary 1993. Wkly Epidemiol Rec 1995:17:121-8.
4. Centers for Disease Control and Prevention. Update: dracunculiasis eradication—Pakistan, 1994. MMWR 1995;44:117-9.
5. WHO. Dracunculiasis update—Sudan. Wkly Epidemiol Rec 1995:7:48-50.
